# Conversion to Stemless Reverse Total Shoulder Arthroplasty After Dislocation of a Stemless Anatomic Implant in a Patient With Persistent Shoulder Instability: Case Report

**DOI:** 10.1155/cro/2476043

**Published:** 2026-01-13

**Authors:** Saif L. Juma, Kamil R. Jarjess, Jamil Haddad, Nicholas David Cominos, Matthew John Yousif

**Affiliations:** ^1^ Department of Osteopathic Medicine, Michigan State University, East Lansing, Michigan, USA, msu.edu; ^2^ Department of Arts and Sciences, Oakland University, Rochester, Michigan, USA, oakland.edu; ^3^ Department of Orthopedic Surgery, McLaren Macomb, Mount Clemens, Michigan, USA; ^4^ Department of Medicine, Wayne State University, Detroit, Michigan, USA, wayne.edu; ^5^ Department of Orthopedic Surgery, Yousif Orthopedic Surgery, Troy, Michigan, USA

## Abstract

Stemless anatomic total shoulder arthroplasty (aTSA) offers bone preservation advantages in younger patients, but postoperative instability requiring revision to reverse total shoulder arthroplasty (rTSA) occurs in 20%–32% of cases, representing a major indication for revision surgery. Although stemless rTSA has demonstrated promising early outcomes in Europe since 2005, it remains investigational and not Food and Drug Administration–approved in the United States. We report a 54‐year‐old female who developed implant dislocation and subscapularis tendon failure 8 weeks after primary stemless aTSA (Sidus system, Zimmer Biomet) for primary glenohumeral osteoarthritis. Given the well‐fixed, convertible stemless humeral component and adequate metaphyseal bone quality, off‐label conversion to stemless rTSA was performed with component retention. This approach is aimed at preserving proximal humeral bone stock, reducing operative time and blood loss, and potentially decreasing infection risk. At 2‐year follow‐up, the patient demonstrated excellent functional outcomes, improved range of motion, and stable radiographic findings. To our knowledge, this is the first report describing clinical and radiographic outcomes following revision of a dislocated stemless anatomic shoulder implant to a stemless reverse configuration.

## 1. Introduction

Shoulder arthroplasty has evolved significantly over the past two decades, with stemless humeral components emerging as an alternative to traditional stemmed designs for both anatomic and reverse configurations [1]. Stemless anatomic humeral implants were first introduced in Europe in 2004 to restore glenohumeral function and subsequently received FDA approval in the United States in 2016, initially with only the Simpliciti model available [2]. Since then, several additional stemless anatomic total shoulder arthroplasty (aTSA) systems, including Sidus, Catalyst, and Arthrosurface, have been introduced [3]. In contrast, stemless humeral implants for reverse total shoulder arthroplasty (rTSA) have been utilized in Europe since 2005 but are only recently undergoing FDA investigational device exemption (IDE) trials in the United States [4]. Despite increasing interest in these designs, clinical and radiographic outcome data for stemless reverse implants, particularly in the revision setting, remain limited in the United States [4].

Traditional stemmed humeral implants have been associated with complications such as intraoperative humeral shaft fracture, stress shielding, loosening, and postoperative periprosthetic fracture [5]. Revision of stemmed implants can be technically challenging, often requiring humeral osteotomy, which increases the risk of intraoperative and postoperative fracture [5]. Wodarek and Shields reported fracture rates of up to 16% associated with stem removal during revision procedures [3]. In contrast, stemless prostheses achieve metaphyseal fixation with minimal bone resection, thereby preserving native bone stock and potentially facilitating easier revision surgery [6].

The safe use of stemless aTSA relies on adequate bone quality and stable metaphyseal fixation to prevent early loosening prior to osseointegration [4]. However, the absence of distal diaphyseal fixation may increase the risk of loosening, and stemless implants have been associated with an increased risk of intraoperative cortical fracture or inadequate fixation in patients with poor bone stock [6, 7]. As a result, careful patient selection is critical, with bone quality and density serving as key determinants of implant suitability [8]. Preoperative computed tomography imaging is commonly used to assess glenoid morphology and assist with surgical planning [9]. Stemless aTSA is typically considered for patients with persistent symptoms despite nonoperative treatment, glenohumeral arthritis, an intact rotator cuff, and adequate humeral bone stock [10].

Although outcomes following stemless aTSA have been increasingly reported since their introduction [4], reports describing conversion of a failed stemless aTSA to a stemless rTSA remain exceedingly rare. To our knowledge, there are no prior reports describing conversion of a dislocated stemless aTSA to a stemless rTSA in a young female patient. In this case report, we describe revision of a dislocated stemless aTSA to a stemless rTSA using the Sidus system (Zimmer Biomet, Warsaw, United States) and report the clinical and radiographic outcomes at 2‐year follow‐up.

## 2. Case Description

A 54‐year‐old Caucasian female presented with chronic, atraumatic right shoulder pain associated with progressive functional limitation and multidirectional instability, refractory to extensive nonoperative management. Her symptoms included persistent pain, stiffness, and limited range of motion that interfered with activities of daily living. Conservative treatments over several years included physical therapy, nonsteroidal anti‐inflammatory medications, and multiple intra‐articular corticosteroid injections, all of which failed to provide durable relief. Her medical history was notable for daily tobacco use, but she had no prior shoulder surgery.

**Figure 1:**
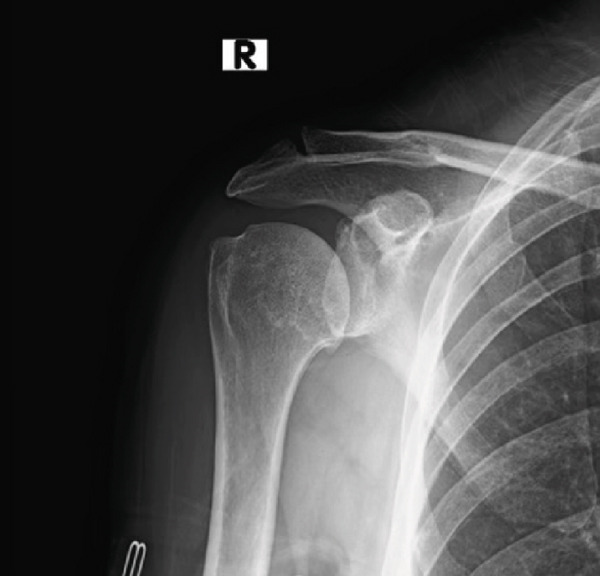
Preoperative shoulder radiograph showing joint space narrowing and osteophyte formation (August 17, 2022).

Initially, a radiographic and ultrasound evaluation of the right shoulder showed glenohumeral joint space narrowing and an intact rotator cuff. Osteophytes are observed along the inferior margin of the humeral head (Figure 1). At the time, the patient had completed extensive nonoperative measures over the course of several years including physical therapy, nonsteroidal anti‐inflammatory drugs, and glenohumeral joint injections. However, she continued to have persistent complaints of right shoulder pain and was interested in surgical intervention.

**Figure 2:**
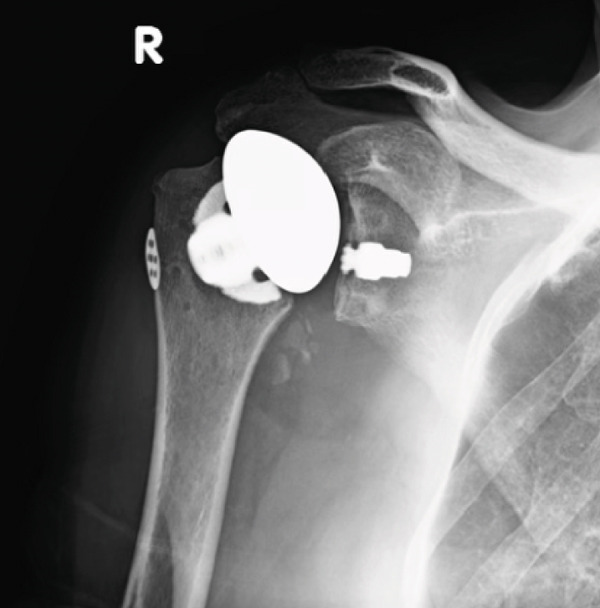
Shoulder radiograph demonstrating a stemless anatomic TSA at first postoperative visit (August 17, 2022).

Preoperative magnetic resonance imaging (MRI) of the right shoulder demonstrated advanced glenohumeral osteoarthritis with associated joint space narrowing and osteophyte formation, without evidence of full‐thickness rotator cuff tearing. Glenoid morphology was appropriate for aTSA, and humeral metaphyseal bone stock was adequate to support stemless fixation. Given the patient′s age, preserved rotator cuff integrity, sufficient metaphyseal bone stock, and absence of humeral bone deficiency, primary stemless aTSA was selected. A stemless design was favored to preserve proximal humeral bone stock and to facilitate potential future revision, particularly in the context of the patient′s tobacco use and the associated risk of impaired soft tissue healing. Accordingly, the orthopedic surgeon proceeded with a stemless aTSA (Figure 2). The risks and benefits of the procedure were discussed in detail during the preoperative visit, with strong emphasis placed on the importance of tobacco cessation.

**Figure 3:**
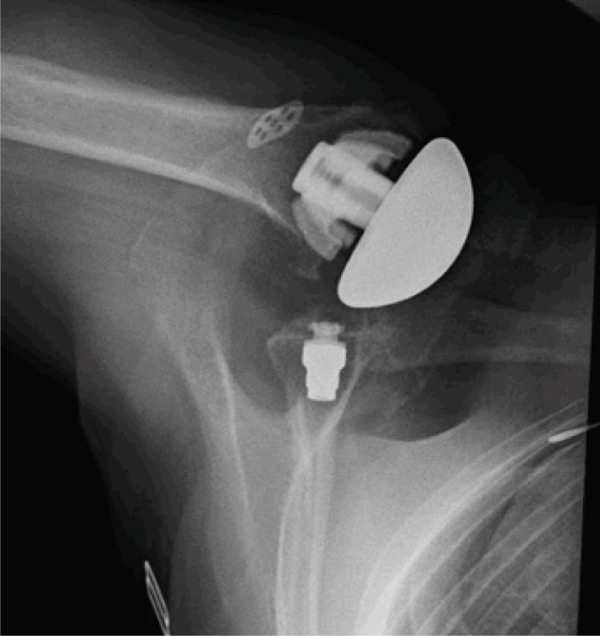
Anteriorly dislocated stemless anatomical glenohumeral implant with well‐fixed humeral and glenoid components (October 5, 2022).

However, 8 weeks after the primary surgical procedure, the patient dislocated her right shoulder and continued to have persistent subluxation (Figure 3). In this case, failure likely occurred due to the patient′s continued tobacco use, which may have contributed to the spontaneous failure of the subscapularis tendon repair.

After dislocated stemless aTSA due to failed subscapularis tendon repair, we proposed conversion to stemless rTSA. This was discussed with the patient and consent for non‐US FDA approved stemless rTSA was obtained. Stemless rTSA was utilized to address the persistent instability since the rotator cuff was severely damaged and to also maintain the benefits of the well‐fixed stemless implant.

**Figure 4:**
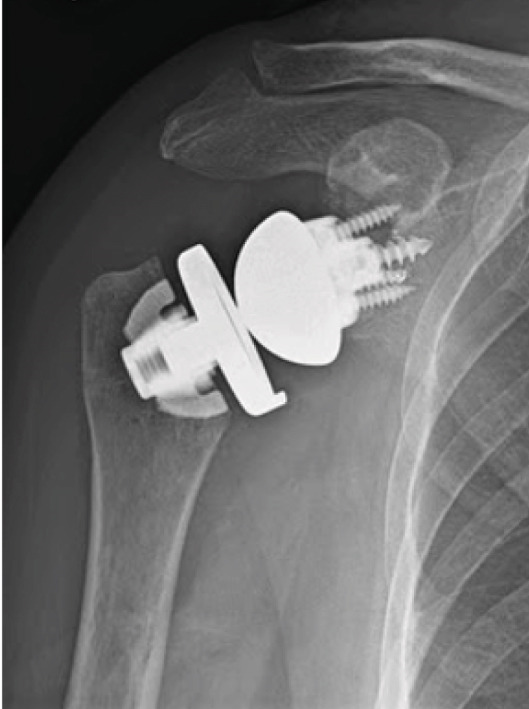
Shoulder radiograph demonstrating a stemless reverse TSA with no signs of loosening at 1‐year postoperative appointment (April 12, 2023).

A deltopectoral incision approximately 10 cm long was made, followed by sharp dissection through the dermis, and blunt dissection through the subcutaneous tissue was made. The previous anatomical humeral head was removed using a tuning fork, osteotome, and mallet. The anchor for the initial total shoulder arthroplasty was intact without signs of loosening or subsidence. Due to a well‐fixed anchor into the proximal humerus, a zimmer press‐fit anchor was placed into the stemless component to conserve bone loss. A standard plus three tray was used. Attention was then turned to the glenoid, where significant synovitis was observed. A complete synovectomy was performed, with the specimen sent for pathological analysis. The existing polyethylene and glenoid components were well‐fixated and were removed using an osteotome and mallet. Following thorough irrigation, the glenoid metal component was removed, and bone grafting was applied throughout the glenoid component. A guide was used to place a central guidewire with a slightly inferior tilt. The central peg hole was drilled, and the glenoid was reamed. A Biomet minibaseplate with miniaugment baseplate was press‐fitted into the glenoid vault, and three screws were placed to achieve excellent fixation. A size 36 standard offset glenosphere was then attached to the metaglene with excellent fixation (Figure 4).

Polyethylene entry components were trialed on the Nanostem, achieving optimal tension, stability, and a full range of motion. Spacer trials were conducted, and the spacer implant was securely fixed onto the stem. The glenohumeral joint was successfully reduced despite a challenging revision due to extensive scar tissue. An anterior capsulotomy was performed, and the remaining subscapularis tendon was meticulously repaired to the humerus using #5 Ethibond sutures. A 4.75 mm bioabsorbable anchor was inserted in the proximal humerus to reinforce the subscapularis tendon, with suture tape whipstitched through the tendon for added stability. The previously placed seven‐hole Synthes button plate and substantial surrounding bursa were removed. The patient emerged from general anesthesia without complications and was transported to the postanesthesia care unit for recovery.

At the 2‐year postoperative follow‐up, the patient reported complete resolution of right shoulder pain. She demonstrated full strength (5/5), with active forward flexion of 170° and abduction of 150°. Radiographs showed no evidence of implant loosening or other complications. The patient was advised to continue a home exercise program and to follow up on an as‐needed basis.

## 3. Discussion

Stemless aTSA has gained increasing attention due to favorable short‐ and mid‐term outcomes in appropriately selected patients. In contrast, reports describing stemless rTSA, particularly in the revision setting, remain limited [11]. This case contributes to the growing but still sparse literature by demonstrating the feasibility and early clinical outcomes of revising a dislocated stemless aTSA to a stemless rTSA while retaining a well‐fixed humeral component.

Importantly, this case should not be interpreted as evidence of the superiority of stemless rTSA over stemmed designs. Rather, it demonstrates that a stemless reverse construct can represent one viable revision option in select circumstances. In revision scenarios where the humeral component is well fixed and conversion to rTSA can be achieved without stem removal, both stemmed and stemless systems may be appropriate. The potential advantage of a stemless design becomes more relevant in situations where humeral component removal is required or where preservation of proximal humeral bone stock is desirable [6, 7, 12].

The rationale for selecting a stemless aTSA as the index procedure in this patient was based on several factors, including her relatively young age, preserved rotator cuff integrity, and adequate metaphyseal bone stock. Stemless designs achieve metaphyseal fixation while minimizing humeral bone resection, which may facilitate future revision if required [6, 7]. At the time of the primary procedure, an anatomic arthroplasty was favored over rTSA to maintain more native shoulder biomechanics in the setting of an intact rotator cuff, consistent with contemporary treatment paradigms [10].

Failure in this case occurred due to postoperative instability associated with subscapularis tendon failure. Subscapularis insufficiency and rotator cuff pathology are well‐recognized causes of instability and revision following aTSA. Parada et al. reported that subscapularis failure and/or rotator cuff tears accounted for 33.9% of revisions and 28.9% of all aTSA complications [12]. In the present case, persistent tobacco use likely contributed to impaired tendon healing and subsequent instability. Prior studies have demonstrated that tobacco use is associated with higher rates of dislocation, readmission, and revision following shoulder arthroplasty [13, 14].

The revision strategy focused on addressing instability while preserving the well‐fixed stemless humeral component. Conversion to rTSA was selected due to irreparable rotator cuff dysfunction, a recognized indication for reverse arthroplasty. Retention of the stemless humeral anchor avoided humeral osteotomy, reduced operative complexity, and preserved bone stock. These considerations are supported by prior reports indicating that removal of stemmed humeral components during revision arthroplasty can increase operative time, blood loss, and the risk of intraoperative fracture [3, 7].

Existing literature on stemless rTSA, largely derived from European cohorts, suggests that stemless reverse designs can achieve clinical and radiographic outcomes comparable with stemmed rTSA in carefully selected patients [6, 11, 15]. Systematic reviews have demonstrated improvements in range of motion and functional scores with low rates of humeral component loosening [6, 11]. However, most published data focus on primary rTSA rather than revision procedures, and evidence specific to revision of failed stemless aTSA remains limited.

Only one prior case report has described revision of a stemless anatomic implant to a stemless reverse configuration with favorable short‐term outcomes [16]. Unlike the present case, that report did not involve revision for instability or dislocation, highlighting the additional technical and clinical challenges addressed here.

This report is limited by its single‐patient design and relatively short follow‐up period. Nevertheless, early radiographic stability and meaningful functional improvement at 2 years are clinically relevant, particularly given that early failures of stemless components typically occur prior to osseointegration [6]. Continued follow‐up is needed to assess long‐term implant durability.

In summary, this case illustrates that stemless rTSA can serve as a feasible revision option following failed stemless aTSA in carefully selected patients with adequate bone stock and irreparable rotator cuff pathology. These findings support stemless rTSA as one potential strategy rather than a superior approach, emphasizing the importance of individualized treatment planning and careful patient selection.

## 4. Conclusion

This case demonstrates that the conversion to a stemless reverse implant can be a viable treatment option for patients with a failed subscapularis tendon repair following aTSA. As stemless aTSA utilization increases, particularly in younger patients, revision procedures are expected to rise, making understanding of all available revision strategies essential for optimal patient outcomes.

## Ethics Statement

Ethical approval was not required for this case report in accordance with institutional policies, as it does not constitute human subjects research. All procedures were conducted in accordance with the principles of the Declaration of Helsinki.

## Consent

Written informed consent was obtained from the patient for the publication of this case report and any accompanying clinical data and imaging. The patient additionally provided informed consent for the off‐label use of a stemless reverse total shoulder arthroplasty implant.

## Disclosure

All authors reviewed and approved the final manuscript.

## Conflicts of Interest

The authors declare no conflicts of interest.

## Author Contributions

Saif L. Juma contributed to manuscript drafting, data interpretation, and literature review. Kamil R. Jarjess contributed to literature review and manuscript editing. Jamil Haddad contributed to surgical care and critical manuscript revision and editing. Nicholas David Cominos contributed to manuscript editing and clinical interpretation. Matthew John Yousif contributed to surgical management, study oversight, and final manuscript revision and editing.

## Funding

No funding was received for this manuscript.

## Data Availability

The data supporting the findings of this study are available from the corresponding author upon reasonable request. Data sharing not applicable to this article as no datasets were generated or analyzed during the current study.
